# Enhancing the Spin Hall Effect of Cylindrically Polarized Beams

**DOI:** 10.3390/mi15030350

**Published:** 2024-02-29

**Authors:** Alexey A. Kovalev, Anton G. Nalimov, Victor V. Kotlyar

**Affiliations:** 1 Image Processing Systems Institute, NRC “Kurchatov Institute”, 151 Molodogvardeyskaya Str., 443001 Samara, Russia; anton@ipsiras.ru (A.G.N.); kotlyar@ipsiras.ru (V.V.K.); 2 Samara National Research University, 34 Moskovskoye Shosse, 443086 Samara, Russia

**Keywords:** spin Hall effect, spin angular momentum, cylindrical vector beam, beam energy, carrier frequency

## Abstract

Two linked gear wheels in a micromachine can be simultaneously rotated in opposite directions by using a laser beam that has in its section areas the spin angular momentum (SAM) of the opposite sign. However, for instance, a cylindrical vector beam has zero SAM in the focus. We alter a cylindrical vector beam so as to generate areas in its focus where the SAM is of opposite signs. The first alteration is adding to the cylindrical vector beam a linearly polarized beam. Thus, we study superposition of two rotationally symmetric beams: those with cylindrical and linear polarization. We obtain an expression for the SAM and prove two of its properties. The first property is that changing superposition coefficients does not change the shape of the SAM density distribution, whereas the intensity changes. The second property is that maximal SAM density is achieved when both beams in the superposition have the same energy. The second perturbation is adding a spatial carrier frequency. We study the SAM density of a cylindrical vector beam with a spatial carrier frequency. Due to periodic modulation, upon propagation in space, such a beam is split into two beams, having left and right elliptic polarization. Thus, in the beam transverse section, areas with the spin of different signs are separated in space, which is a manifestation of the spin Hall effect. We demonstrate that such light beams can be generated by metasurfaces, with the transmittance depending periodically on one coordinate.

## 1. Introduction

Elements of micromachines can be driven by light, including structured light with phase and/or polarization singularities. Thus, of great interest are light fields with orbital and spin angular momentum (OAM and SAM). The SAM causes particles to rotate around their centers of mass [1], and engineering the SAM density can manipulate an ensemble of particles simultaneously. For generating a light field with a specific distribution of the SAM density, the initial field does not necessarily carry nonzero SAM. The optical spin Hall effect is manifested when a zero-SAM beam with complete linear polarization acquires non-zero SAM density as it propagates in space [2,3]. In addition to the spin Hall effect, the optical Hall effect can also be orbital [4,5] or spin-orbital [6].

The spin Hall effect can be radial or angular. The radial spin Hall effect was described in [7], where the authors showed that when a radially polarized optical vortex is focused, the longitudinal component of the SAM density vector has different signs at different distances from the optical axis in the focal plane. The angular spin Hall effect is demonstrated in many of our recent works, for instance in [8], where a high-order cylindrical vector beam is tightly focused and near the focal plane; areas with different signs of SAM density vector reside at different polar angles. Separation of left and right circular polarization in the tight focus was also shown for several other types of vector beams [9,10]. As was found in [10], the spin Hall effect arises in the tight focus of a linearly polarized optical vortex. Such a beam can be easily generated without a metasurface [11] by employing a spatial light modulator.

In our work [12,13], we have found that the spin Hall effect can also be achieved in paraxial vector beams that are not tightly focused. The mechanism of the spin Hall effect is much similar in paraxial light beams and in focus, but in paraxial beams, the orbital Hall effect cannot arise since the longitudinal component of the electric field is negligibly small, whereas in a focus it becomes significant and, thus, transverse energy flows are generated, rotating in opposite directions (orbital Hall effect). In [12], we studied a Gaussian beam with several polarization singularities residing on a ring with a center on the optical axis. Such a beam can be constructed as a superposition of a radially polarized single-ringed Laguerre-Gaussian (LG) beam with a linearly polarized Gaussian beam. In [13], we investigated a coaxial superposition of two paraxial single-ringed LG beams with topological charges (TC) *n* and −*n* and linearly polarized along the horizontal axis, and of two LG beams with the TCs *m* and −*m* and linearly polarized along the vertical axis.

The work [12] shows that perturbation of a cylindrical vector beam by superposing a Gaussian beam, or other types of perturbations, can give rise to the spin Hall effect. The above-listed works do not consider the propagation of a paraxial vector beam with one-dimensional periodical modulation, and the longitudinal component of the SAM vector is not calculated for such a beam. In addition, these works do not study the generation of two vortex Gaussian beams with left and right circular polarization from a vector field with one-dimensional periodical modulation.

In contrast to [10], generating light fields like those from [9,12,13] is more challenging since it requires modulating not only the phase distribution but polarization as well. To achieve this goal, metasurfaces can be employed. Separation of left-hand and right-hand elliptically polarized components of a light field by using a metasurface was implemented for the first time in work [14]. In this work, propagation of a near-infrared (1.2–1.7 μm) light field was studied through a metasurface composed of periodic gold V-shape nanoantennas. In the far field, the third component of the Stokes vector was measured. It was demonstrated that two areas were generated at the edges of the light beam, where the Stokes component had different signs. Thus, areas with left and right elliptic polarization are present in the beam. In [15,16], a metalens was used to enhance the transverse shift of beams with left and right circular polarizations. For example, in [16], for enhancing the Hall effect, subwavelength diffraction grating was used. However, the beams with opposite spin were shifted along grating lines. In [17], for the wavelength of 633 nm and by using a q-plate, a vortex beam with linear polarization was split into a Gaussian beam with left circular polarization and a vortex beam with right circular polarization. In works [18,19,20,21,22,23], metasurface-based polarization converters were studied for millimeter-wave light. For instance, in [20], a metasurface composed of metallic stripes is illuminated by linearly polarized millimeter-wave irradiation, and two beams with left and right circular polarization are reflected by angles of 30 degrees. In [21], a reflective metasurface was computed for the millimeter range, intended for converting a field with linear polarization into two fields with left-hand and two fields with right-hand circular polarization. A similar metasurface, but for demonstrating the spin Hall effect at transmission, is considered in [22]. In works [24,25], metasurfaces are investigated for converting the polarization of an infrared radiance, whereas in [26,27,28]—for visible light. We note that the above-listed works do not study the theoretical propagation of light after passing the metalenses.

The spin Hall effect in the above-mentioned works was investigated both in paraxial beams and in the focus, but it manifested itself poorly, i.e., in the beam section, areas with opposite-sign spins were generated, but the spin angular momentum density had a low magnitude, i.e., the polarization ellipses in these areas are strongly elongated. Therefore, a problem arises in finding such initial distributions of linear polarization that would enhance the Hall effect and make the polarization ellipse more circular. Vector light fields are usually generated by using SLM and q-plates, but it is more convenient to combine all the elements into one implemented as a metasurface.

In this paper, we investigate two perturbations of a cylindrical vector beam, i.e., we induce two different asymmetries into this beam, that can raise the spin Hall effect. At first, we study the superposition of general-shape cylindrically and linearly polarized beams. Thus, the first constituent beam can have radial, azimuthal, saddle [29], or another cylindrical polarization [30]. The second beam should have linear polarization in an arbitrary direction, and both beams can have an arbitrary rotationally-symmetric shape, i.e., they can be Laguerre-Gaussian [31], Bessel-Gaussian [32], Hypergeometric-Gaussian beams [33], or some more exotic beams [34] with rotational symmetry. We prove that the spin Hall effect is the strongest, i.e., the spin angular momentum density is maximal, when both constituent beams in the superposition have the same energy. The second perturbation of a paraxial cylindrical vector beam is adding a spatial carrier frequency. This frequency splits the beam into two, and we implemented it with a metalens. An expression is obtained for the longitudinal component of the spin angular momentum density at an arbitrary distance from the waist plane. We also show that such a beam has linear inhomogeneous polarization in the initial plane, but, after propagation in space, two beams are generated, one with left circular polarization and another with right circular polarization, i.e., the spin Hall effect manifests itself almost fully.

## 2. Spin Angular Momentum of a Superposition of Rotationally Symmetric Beams with Cylindrical and Linear Polarization

We consider here the superpositions of two paraxial vector rotationally symmetric light fields, one with cylindrical polarization and the other with linear polarization:

(1) $$E\left(r,\varphi ,z\right)=C_{C}E_{C}\left(r,\varphi ,z\right)+C_{L}E_{L}\left(r,\varphi ,z\right),$$

with (*r*, *φ*, *z*) being the cylindrical coordinates, *C_C_* and *C_L_* being the superposition coefficients, defining the contributions of the cylindrically and linearly polarized beams.

In the initial plane *z* = 0, the complex amplitudes of both of these fields are given by

(2) $$E_{C}\left(r,\varphi ,0\right)=A\left(r\right)\left[\begin{array}{c} \cos\left(m\varphi +\alpha \right)\\ \cos\left(m\varphi +\beta \right) \end{array}\right],$$

(3) $$E_{L}\left(r,\varphi ,0\right)=B\left(r\right)\left[\begin{array}{c} \cos\gamma \\ \sin\gamma \end{array}\right].$$

In Equations (2) and (3), *A*(*r*) and *B*(*r*) are real-valued functions, *m* is the order of cylindrical polarization, *α* and *β* define its azimuthal angle [30], and *γ* defines the orientation of linear polarization.

Paraxial propagation in free space is described by the well-known Fresnel transform, which in polar coordinates is given by

(4) $$E\left(r,\varphi ,z\right)=\frac{-ik}{2\pi z}\int_{0}^{\infty }\int_{0}^{2\pi }E\left(\rho ,\theta ,0\right)\exp\left\{\frac{ik}{2z}\left[r^{2}+\rho^{2}-2r\rho \cos\left(\theta -\varphi \right)\right]\right\}\rho d\rho d\theta .$$

For a scalar vortex field in the form *E*_V_(*ρ*, *θ*, 0) = *G*(*ρ*)exp(*imθ*), the integral over the angle *θ* is expressed via the Bessel function, and the Fresnel transform reads as

(5) $$E_{V}\left(r,\varphi ,z\right)={\left(-i\right)}^{m+1}\frac{k}{z}\exp\left(\frac{ikr^{2}}{2z}+im\varphi \right)\int_{0}^{\infty }G\left(\rho \right)\exp\left(\frac{ik\rho^{2}}{2z}\right)J_{m}\left(\frac{kr\rho }{z}\right)\rho d\rho .$$

Decomposing the cylindrically polarized field (2) into two optical vortices of the orders ±*m* and using the auxiliary integral (5), we obtain the following complex amplitude of the fields (2) and (3) at a distance *z*:

(6) $$\begin{array}{l} E_{C}\left(r,\varphi ,z\right)={\left(-i\right)}^{m+1}\frac{k}{z}\exp\left(\frac{ikr^{2}}{2z}\right)\\ \;\;\;\;\times \left[\int_{0}^{\infty }A\left(\rho \right)\exp\left(\frac{ik\rho^{2}}{2z}\right)J_{m}\left(\frac{kr\rho }{z}\right)\rho d\rho \right]\left[\begin{array}{c} \cos\left(m\varphi +\alpha \right)\\ \cos\left(m\varphi +\beta \right) \end{array}\right], \end{array}$$

(7) $$E_{L}\left(r,\varphi ,z\right)=-i\frac{k}{z}\exp\left(\frac{ikr^{2}}{2z}\right)\left[\int_{0}^{\infty }B\left(\rho \right)\exp\left(\frac{ik\rho^{2}}{2z}\right)J_{0}\left(\frac{kr\rho }{z}\right)\rho d\rho \right]\left[\begin{array}{c} \cos\gamma \\ \sin\gamma \end{array}\right].$$

In a paraxial light beam, only the longitudinal component of the spin angular momentum vector is significant. In general, it is equal to

(8) Sz=2ImEx*Ey.

For a sum of two fields, given by Equation (1), the SAM density is equal to

(9) Sz=2ImCCEC,x+CLEL,x*CCECy+CLELy        =CC2SC,z+CL2SL,z+2CCCLImEC,x*EL,y+EL,x*EC,y,

where *S*_C,*z*_ and *S*_L,*z*_ are separate SAM densities of the cylindrically and of the elliptically polarized beams. They are zero, and thus only the third term remains in Equation (9). Substituting here the field components from Equations (6) and (7), we get

(10) Sz=2CCCLkz2sinγcosmφ+α−cosγcosmφ+β    ×Imim∫0∞Aρexp−ikρ22zJmkrρzρdρ    ×∫0∞Bσexpikσ22zJ0krσzσdσ.

It is seen that the SAM density is, in general, nonzero. It is also seen that the transverse shape of the SAM density distribution depends on the shape of both constituent beams but is independent of their weights in the superposition, i.e., changing the weights changes the SAM density distribution only in magnitude but not in its shape. Equation (10) also reveals that the SAM density becomes zero on rings when either the *E_x_* or *E_y_* component becomes zero, or the expression in the second line of Equation (10) becomes real.

## 3. Energies of Cylindrically and Linearly Polarized Beams for Maximizing the Spin Angular Momentum Density

Thus, it is seen that, despite neither the cylindrically nor linearly polarized fields acquiring spin angular momentum upon propagation in free space, their superposition does. We aim to find the weights of the constituent beams in the superposition that yield the maximal SAM. It stands to reason that the SAM density in Equation (10) grows with both the coefficients *C*_C_ and *C*_L_. But it follows that the energy of beam (1) grows as well. Thus, we should fix the energy at some value, e.g., *W*_0_. The energy of an arbitrary paraxial vector field can be obtained as

(11) $$W=\int_{0}^{\infty }\int_{0}^{2\pi }\left[{\left|E_{x}\left(r,\varphi ,z\right)\right|}^{2}+{\left|E_{y}\left(r,\varphi ,z\right)\right|}^{2}\right]rdrd\varphi .$$

The energy of the cylindrically polarized beam (2) equals

(12) $$W_{C}=\int_{0}^{\infty }A^{2}\left(r\right)rdr\int_{0}^{2\pi }\left[\cos^{2}\left(m\varphi +\alpha \right)+\cos^{2}\left(m\varphi +\beta \right)\right]d\varphi =2\pi \int_{0}^{\infty }A^{2}\left(r\right)rdr,$$

whereas the energy of the linearly polarized beam (3) equals

(13) $$W_{L}=2\pi \int_{0}^{\infty }B^{2}\left(r\right)rdr.$$

The fields (2) and (3) consist of independent OAM components of the orders ±*m* and 0. Thus, the full energy of the beam (1) can be obtained as the sum:

(14) $$W=C_{C}^{2}W_{C}+C_{L}^{2}W_{L}.$$

Since the SAM density is proportional to the product *C*_C_*C*_L_, it can be maximized at an arbitrary point (*r*, *φ*, *z*) for all superpositions (1) with fixed energy *W*_0_ by solving the following optimization problem:

(15) $$\left\{\begin{array}{l} C_{C}C_{L}\to \max,\\ C_{C}^{2}W_{C}+C_{L}^{2}W_{L}=W_{0}. \end{array}\right.$$

The Lagrangian function for this problem reads as

(16) $$L\left(C_{C},C_{L}\right)=C_{C}C_{L}+\lambda_{0}\left(C_{C}^{2}W_{C}+C_{L}^{2}W_{L}-W_{0}\right),$$

where *λ*_0_ is the Lagrange multiplier.

Differentiation by *C*_C_ and *C*_L_ yields two equations:

(17) $$\left\{\begin{array}{l} C_{L}+2\lambda_{0}C_{C}W_{C}=0,\\ C_{C}+2\lambda_{0}C_{L}W_{L}=0. \end{array}\right.$$

Multiplying the first and second equations by *C*_C_ and *C*_L_, respectively, yields

(18) $$C_{C}^{2}W_{C}=C_{L}^{2}W_{L}=-\frac{C_{C}C_{L}}{2\lambda_{0}}.$$

This means that the energies of both beams in the superposition should be equal to each other and to *W*_0_/2. We note that we obtained a similar result (18) earlier for a particular case [12] of a superposition of a cylindrically polarized single-ringed LG beam with a linearly polarized Gaussian beam. In the current work, we generalize the results of the work [12] for a similar superposition, but with arbitrary amplitudes *A*(*r*) and *B*(*r*) in Equations (2) and (3).

## 4. Intensity and Spin Density of a Gaussian Vector Field with One-Dimensional Periodical Modulation

In this section, we analyze the propagation of a paraxial Gaussian vector beam with the following initial Jones vector:

(19) $$E(x,y)=\left(\begin{array}{l} \cos(\alpha x)\\ \sin(\alpha x) \end{array}\right)\exp\left(-\frac{x^{2}+y^{2}}{w^{2}}\right),$$

where (*x,y*) are the Cartesian coordinates in the beam cross-section, *w* is the waist radius of the Gaussian beam, *α* = 2π/*d* is the inverse period or spatial frequency of the field amplitude, and *d* is the period. The light field can be called the vector field with one-dimensional periodic modulation. The field (19) can be represented as the superposition of a left circularly polarized wave and a right circularly polarized wave:

(20) $$\left(\begin{array}{l} \cos(\alpha x)\\ \sin(\alpha x) \end{array}\right)e^{-{\left(r/w\right)}^{2}}=\frac{1}{2}\left[e^{i\alpha x}\left(\begin{array}{l} 1\\ -i \end{array}\right)+e^{-i\alpha x}\left(\begin{array}{l} 1\\ i \end{array}\right)\right]e^{-{\left(r/w\right)}^{2}},$$

with $r^{2}=x^{2}+y^{2}$. In the initial plane, the intensity of light field (19) is described by that of the Gaussian beam:

(21) $$I_{0}={\left|E_{x}\right|}^{2}+{\left|E_{y}\right|}^{2}=\exp\left(-2\frac{x^{2}+y^{2}}{w^{2}}\right).$$

The Jones vector of field (19) at an arbitrary propagation distance *z* from the initial plane is given by

(22) $$\begin{array}{l} E(x,y,z)=-i\left(\frac{z_{0}}{2zq(z)}\right)\exp\left(ik\frac{r^{2}}{2z}-{\left(\frac{z_{0}y}{zw}\right)}^{2}\frac{1}{q(z)}\right)\times \\ \left[\exp\left(-\frac{1}{q(z)}{\left(\frac{z_{0}x}{zw}-\frac{\alpha w}{2}\right)}^{2}\right)\left(\begin{array}{l} 1\\ -i \end{array}\right)+\exp\left(-\frac{1}{q(z)}{\left(\frac{z_{0}x}{zw}+\frac{\alpha w}{2}\right)}^{2}\right)\left(\begin{array}{l} 1\\ i \end{array}\right)\right], \end{array}$$

where $q(z)=1-iz_{0}/z$ and $z_{0}=kw^{2}/2$ is the Rayleigh distance and *k* is the wavenumber of light. As seen from Equation (22), upon free-space propagation, the Gaussian beam (21) consists of two Gaussian beams, shifted along the *x*-axis and having left and right circular polarization. The centers of both beams are shifted from the optical axis, being located at points $x_{\pm }=\pm \left(\alpha z/k\right)$. If the shift is much larger than the diameter of the beams, then the intensity of vector field (22) is as follows:

(23) $$I(x,y,z)={\left(\frac{z_{0}}{z\left|q(z)\right|}\right)}^{2}\exp\left(-2{\left(\frac{z_{0}r}{zw\left|q(z)\right|}\right)}^{2}-{\left(\frac{\alpha w}{\sqrt{2}\left|q(z)\right|}\right)}^{2}\right)\cosh\left(\frac{2\alpha xz_{0}}{z{\left|q(z)\right|}^{2}}\right).$$

For a paraxial field, the SAM density has no transverse components. Thus, the longitudinal component of the spin angular momentum (SAM) [35] of field (22) can be derived in the form

(24) Sz=2ImEx*Ey=−z0zq(z)2exp−2z0rzwq(z)2−12αwq(z)2sinh2αxz0zq(z)2.

The spin density normalized by the intensity is given by

(25) $$S_{z}/I=-\tanh\left(\frac{2\alpha xz_{0}}{z{\left|q(z)\right|}^{2}}\right).$$

This indicates that the spin of the initial plane, field (19), is zero (*S_z_* = 0), but as light field (22) propagates in free space, two areas of opposite-sign spin are generated, where the normalized spin density is given by (25). This is the simplest way to demonstrate the spin Hall effect and to obtain two opposite-handed circularly polarized focal regions from linearly polarized light.

Circular polarization is at points *x* where the condition $S_{z}=\pm I$ is fulfilled. This, however, can occur only when the argument of the hyperbolic tangent in Equation (25) tends to plus or minus infinity. With the normalized spin density in (25) depending on the propagation distance *z* from the waist, its maximum is achieved at the Rayleigh distance *z* = *z*_0_.

For generating the initial light field (19), a metasurface can be used, which is described by a polarization transformation matrix that rotates the linear polarization vector of the incident field by an angle of *θ* = *αx*, since the following identity takes place:

(26) $$\left(\begin{array}{l} \cos(\alpha x)\;\;-\sin(\alpha x)\\ \sin(\alpha x)\;\;\;\;\;\cos(\alpha x) \end{array}\right)\left(\begin{array}{l} 1\\ 0 \end{array}\right)=\left(\begin{array}{l} \cos(\alpha x)\\ \sin(\alpha x) \end{array}\right).$$

As seen from (26), such a metasurface rotates the linear polarization of the incident beam by an angle periodically depending on the horizontal coordinate *x*.

## 5. Cylindrical Vector Beam with Spatial Carrier Frequency

The light field (19) can be generalized so that upon propagation in free space, it would generate not only the spin Hall effect but also the orbital one. To do this, we consider the initial field with the following Jones vector:

(27) $$E_{n}(x,y)=\left(\begin{array}{l} \cos(n\varphi +\alpha x)\\ \sin(n\varphi +\alpha x) \end{array}\right)\exp\left(-\frac{x^{2}+y^{2}}{w^{2}}\right),$$

where *φ* is the azimuthal angle in the beam transverse section, tan(*φ*) = *y*/*x*, and *n* is an integer number defining the order of the vector field. The field of (27) can be represented as a sum of two fields with right- and left-handed circular polarizations:

(28) $$\left(\begin{array}{l} \cos(n\varphi +\alpha x)\\ \sin(n\varphi +\alpha x) \end{array}\right)e^{-{\left(r/w\right)}^{2}}=\frac{1}{2}\left[e^{i(n\varphi +\alpha x)}\left(\begin{array}{l} 1\\ -i \end{array}\right)+e^{-i(n\varphi +\alpha x)}\left(\begin{array}{l} 1\\ i \end{array}\right)\right]e^{-{\left(r/w\right)}^{2}}.$$

At *α* = 0, the field of (28) reduces to an *n*th-order cylindrical vector field [30]. Therefore, for a nonzero value of *α*, the field of (28) can be called a cylindrical vector field (beam) with a spatial carrier frequency. At an arbitrary propagation distance *z* from the waist plane, the field of (27) is given by

(29) $$\begin{array}{l} E_{n}=E_{n-}\left(\begin{array}{l} 1\\ -i \end{array}\right)+E_{n+}\left(\begin{array}{l} 1\\ i \end{array}\right),\\ E_{n\pm }(x,y,z)=-\frac{i^{n+1}}{2}\left(\frac{z_{0}}{zq(z)}\right)\sqrt{\frac{\pi Q_{\pm }}{2}}\exp\left(\mp in\psi_{\pm }+i\frac{kr^{2}}{2z}-Q_{\pm }\right)\times \\ \times \left[I_{(n-1)/2}(Q_{\pm })-I_{(n+1)/2}(Q_{\pm })\right],\\ q(z)=1-i\frac{z_{0}}{z},\;\tan\psi_{\pm }=\frac{y}{x\pm \frac{\alpha z}{k}},\\ Q_{\pm }=\frac{{\left(\alpha w\right)}^{2}}{8q(z)}+{\left(\frac{z_{0}r}{\sqrt{2}zw}\right)}^{2}\frac{1}{q(z)}\pm \frac{\alpha z_{0}x}{2zq(z)}. \end{array}$$

The functions *I_v_*(*x*) in Equation (29) are modified Bessel functions. According to Equation (29), the paraxial vector field is composed of two off-axis vortex beams with right- and left-handed circular polarizations, with centers of phase singularities (vortex centers) found at points $x_{\pm }=\pm \left(\alpha z/k\right)$. The phase singularity points and the intensity nulls of field (29) coincide. Optical vortices near these points have the opposite-sign topological charges, *n* and −*n*. Near the intensity nulls, each component of the light field is, respectively, defined by the amplitude ${\left(x+\alpha -iy\right)}^{n}$ and ${\left(x-\alpha +iy\right)}^{n}$. We note that if *n* = 0, then field (29) reduces exactly to field (22). In the initial plane, field (27) has neither orbital angular momentum (OAM) nor spin density. However, upon free-space propagation, the single field splits into two fields, both having the opposite-sign longitudinal SAM and the OAM. In the areas of negative spin (left-handed circular polarization), both the topological charge and the OAM are positive, and vice versa, where the spin is positive (right-handed circular polarization), both the OAM and the topological charge are negative. The total spin and OAM of the whole field remain equal to zero, as is the case for the initial field (27).

**Figure 1:**
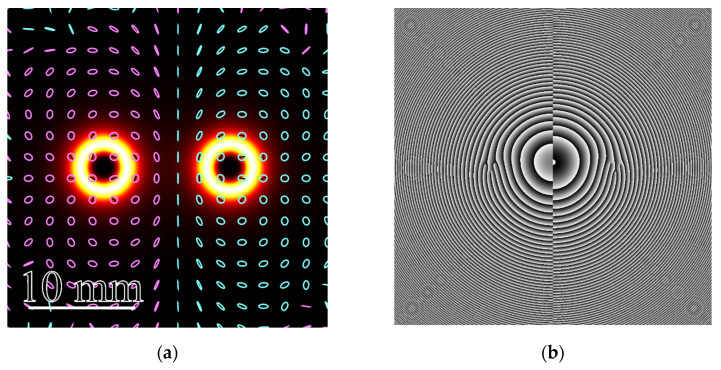
Intensity distribution of beam (27) with *w* = 1 mm, *n* = 3, and α = 0.001*k* at a distance of *z*_0_ from the waist plane, shown by white-yellow rings (**a**), and polarization distribution over the beam transverse section, shown by ellipses (pink ellipses denote right-handed polarization *S_z_* > 0 and cyan ellipses denote left-handed polarization *S_z_* < 0); phase distribution of one transverse component of the light field *E_x_* (**b**). The size of both figures is 30 × 30 mm.

Figure 1 illustrates the intensity distribution (white-yellow rings) (Figure 1a) and phase distribution of the *E_x_* component (Figure 1b) of beam (27) with *w* = 1 mm, *n* = 3, and α = 0.001*k* at distance *z*_0_ from the waist plane. All distributions are obtained by using a Fresnel transform for the wavelength 532 nm. The polarization distribution pattern in Figure 1a is shown by pink (*S_z_* > 0) and cyan (*S_z_* < 0) ellipses. As seen in Figure 1a, the intensity distribution contains two bright rings, with near-circular polarization inside these rings. Besides, the left and right patterns show opposite-sign polarization (right-handed circular polarization near the left ring and left-handed circular polarization near the right ring). On the right of Figure 1b, three screw dislocations are seen, confirming that the topological charge of the right ring in the field *E_x_* is equal to *n* = 3. On the left of Figure 1b, the phase distribution contains three screw dislocations of opposite sign, confirming that the left ring of the field *E_x_* has a topological charge of *n* = −3.

## 6. Numerical Simulation of Superpositions of Rotationally Symmetric Beams with Cylindrical and Linear Polarization

The case when the cylindrically and linearly polarized beams are, respectively, a single-ringed Laguerre-Gaussian beam and a Gaussian beam has been investigated [12]. Thus, here we may consider some other paraxial beams. For instance, we can construct a cylindrically polarized beam with several light rings as the superposition of two Laguerre-Gaussian beams of opposite orders with nonzero radial index and opposite circular polarizations, combining them with a linearly polarized Gaussian beam of different waist radius. The complex amplitude of the cylindrically polarized Laguerre-Gaussian beam in the initial plane is given by

(30) $$E_{C}\left(r,\varphi ,z=0\right)=W_{\mathrm{LG}}^{-1/2}{\left(\frac{r}{w_{0}}\right)}^{m}L_{p}^{m}\left(\frac{2r^{2}}{w_{0}^{2}}\right)\exp\left(-\frac{r^{2}}{w_{0}^{2}}\right)\left[\begin{array}{c} \cos\left(m\varphi \right)\\ \sin\left(m\varphi \right) \end{array}\right],$$

where *w*_0_ is the waist radius, *m* and *p* are, respectively, the azimuthal and radial indices defining the topological charge and the number of rings, $L_{p}^{m}(x)$ is the Laguerre polynomial, and *W*_LG_ is the normalizing factor equal to the energy of the Laguerre-Gaussian beam and introduced to ensure that the energy of beam (30) is unit:

(31) $$W_{\mathrm{LG}}=\frac{\pi w_{0}^{2}}{2^{m+1}}\frac{\left(m+p\right)!}{p!}.$$

We suppose that the linearly polarized Gaussian beam is horizontally polarized, and thus, it has the following complex amplitude:

(32) $$E_{L}\left(r,\varphi ,z=0\right)=W_{G}^{-1/2}\exp\left(-\frac{r^{2}}{w_{1}^{2}}\right)\left[\begin{array}{c} 1\\ 0 \end{array}\right],$$

where *w*_1_ is the waist radius that should be greater than *w*_0_ so that the Gaussian beam overlaps the Laguerre-Gaussian beam, and *W*_G_ is the normalizing factor for reducing the energy of beam (32) to a unit value:

(33) $$W_{\mathrm{LG}}=\frac{\pi w_{1}^{2}}{2}.$$

**Figure 2:**
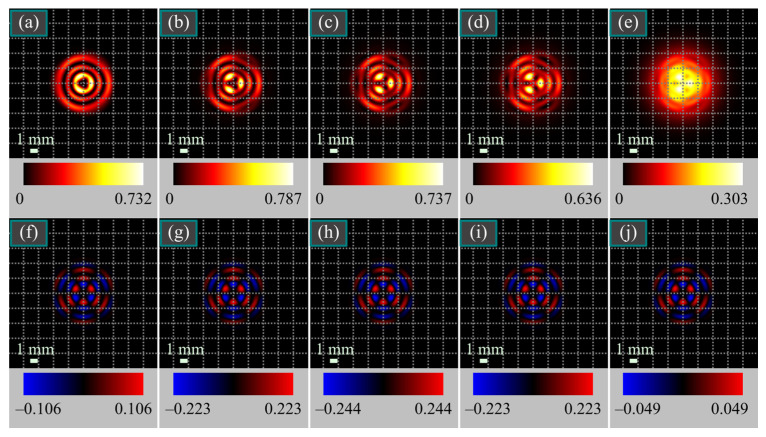
Intensity (**a**–**e**) and SAM density (**f**–**j**) distributions of several superpositions of the cylindrically polarized Laguerre-Gaussian beams (30) and linearly polarized Gaussian beams (32) with different weight coefficients for the following parameters: wavelength λ = 532 nm, Gaussian beam waist radii *w*_0_ = 1 mm and *w*_1_ = 5 mm, radial and azimuthal orders of the cylindrically polarized Laguerre-Gaussian beam *p* = 2 and *m* = 3, propagation distance from the initial plane *z* = *z*_0_, superposition coefficients *C*_C_^2^ = 0.95, *C*_L_^2^ = 0.05 (**a**,**f**), *C*_C_^2^ = 0.70, *C*_L_^2^ = 0.30 (**b**,**g**), *C*_C_^2^ = *C*_L_^2^ = 0.50 (**c**,**h**), *C*_C_^2^ = 0.30, *C*_L_^2^ = 0.70 (**d**,**i**), and *C*_C_^2^ = 0.01, *C*_L_^2^ = 0.99 (**e**,**j**). The numbers near the color scales denote the minimal and maximal values.

Figure 2 illustrates the intensity and SAM density distributions of several superpositions of light beams (30) and (32) with different weight coefficients.

Figure 2 confirms that the maximal SAM density is achieved when the energies of both constituent beams are the same, i.e., *C*_C_ = *C*_L_. It also confirms that the SAM density changes with the weight coefficients only by magnitude rather than shape.

Another example of the composite light field in (1) is when a cylindrically polarized beam is constructed as the superposition of two Bessel-Gaussian beams [32] of the orders ±*m* and combined with a ring-shaped non-vortex beam. The complex amplitude of the cylindrically polarized Bessel-Gaussian beam in the initial plane is given by

(34) $$E_{C}\left(r,\varphi ,z=0\right)=W_{\mathrm{BG}}^{-1/2}J_{m}\left(\alpha_{0}r\right)\exp\left(-\frac{r^{2}}{w_{0}^{2}}\right)\left[\begin{array}{c} \cos\left(m\varphi \right)\\ \sin\left(m\varphi \right) \end{array}\right],$$

where *w*_0_ is the waist radius, *m* is the order of cylindrical polarization, α_0_ is the scaling factor of the Bessel-Gaussian beam defining the radius of the light ring, *J_m_*(*x*) is the *m*th-order Bessel function of the first kind, and *W*_BG_ is the normalizing factor for reducing the energy of beam (34) to a unit value:

(35) $$W_{\mathrm{BG}}=\frac{\pi w_{0}^{2}}{2}\exp\left[-{\left(\frac{\alpha_{0}w_{0}}{2}\right)}^{2}\right]I_{m}\left[{\left(\frac{\alpha_{0}w_{0}}{2}\right)}^{2}\right],$$

with *I_m_*(ξ) being the modified *m*th-order Bessel function.

In order to construct a non-vortex single-ringed light beam, we can subtract two Gaussian beams with different waist radii *w*_01_ and *w*_02_:

(36) $$E_{L}\left(r,\varphi ,z=0\right)=W_{\mathrm{DG}}^{-1/2}\left[\exp\left(-\frac{r^{2}}{w_{01}^{2}}\right)-\chi \exp\left(-\frac{r^{2}}{w_{02}^{2}}\right)\right]\left[\begin{array}{c} 1\\ 0 \end{array}\right],$$

where the coefficient *χ* is chosen so that the field has zero intensity on the optical axis at some distance *z*: *χ* = *q*_2_/*q*_1_ with *q_i_* = 1 + *i*λ*z*/(π*w*^2^_0*i*_), *i* = 1, 2, and *W*_BG_ is the normalizing factor for reducing the energy of beam (36) to a unit value:

(37) WDG=πw0122+πw0222χ2−2πw012w022w012+w022Reχ.

**Figure 3:**
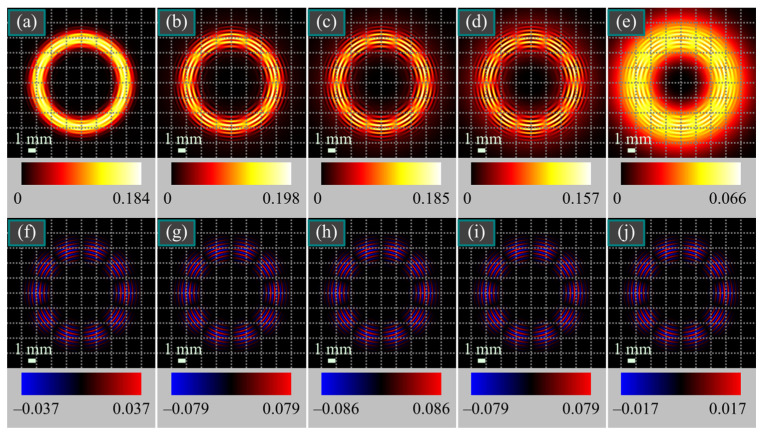
Intensity (**a**–**e**) and SAM density (**f**–**j**) distributions of several superpositions of the cylindrically polarized Bessel-Gaussian beams (34) and linearly polarized difference of two Gaussian beams (36) with different weight coefficients for the following parameters: wavelength λ = 532 nm, waist radius of the Gaussian envelope of the Bessel-Gaussian beam *w*_0_ = 1 mm, scaling factor α_0_ = *k*/1000, order of cylindrical polarization *m* = 5, waist radii of the subtracted linearly polarized Gaussian beams *w*_01_ = 5 mm and *w*_02_ = 7 mm (at these radii the light ring of the difference beam has the same radius as that of the Bessel-Gaussian beam), propagation distance from the initial plane *z* = *z*_0_, superposition coefficients *C*_C_^2^ = 0.95, *C*_L_^2^ = 0.05 (**a**,**f**), *C*_C_^2^ = 0.70, *C*_L_^2^ = 0.30 (**b**,**g**), *C*_C_^2^ = *C*_L_^2^ = 0.50 (**c**,**h**), *C*_C_^2^ = 0.30, *C*_L_^2^ = 0.70 (**d**,**i**), and *C*_C_^2^ = 0.01, *C*_L_^2^ = 0.99 (**e**,**j**). The numbers near the color scales denote the minimal and maximal values.

Figure 3 depicts the intensity and SAM density distributions of several superpositions of light beams (34) and (36) with different weight coefficients.

Figure 3 also confirms that in this case, when both beams in superposition (1) are different from those in Figure 2, the maximal SAM density is still achieved when the energies of both constituent beams are the same, i.e., *C*_C_ = *C*_L_. As shown in Figure 2, the SAM density changes with the weight coefficients only by magnitude rather than shape.

## 7. Designing a Metalens for Generating Two Beams with the Opposite-Sign Spins

In this section, we demonstrate how the vector beams (19) and (27) can be generated by using only one optical element. We designed this element using a method described earlier in [11]. Such a metasurface [11] is composed of subwavelength binary gratings, each of which rotates the polarization vector by a given angle. We note that the spin Hall effect was observed in work [17] by generating a beam using an SLM and q-plates. Such a setup requires an exact adjustment. In addition, the opposite-sign spin in [17] was generated in the focus and in the ring that surrounds it. In the current work, we generate the opposite-sign spin in two similar off-axis foci or in two similar off-axis rings. This is more convenient for separate use of the generated beams.

As we earlier noted, field (19) can be generated by using a metasurface in (26). The metasurface should have the following matrix describing the polarization transformation of the incident vector field:

(38) $$\hat{R}(x)=\left(\begin{array}{l} \cos\alpha x\;-\sin\alpha x\\ \sin\alpha x\;\;\;\cos\alpha x \end{array}\right),$$

where α is inversely proportional to the metasurface structure period along the *x*-axis. If the incident field is a plane wave linearly polarized along the *x*-axis, then the polarization direction of the outgoing beam depends on the coordinate *x* (26).

**Figure 4:**
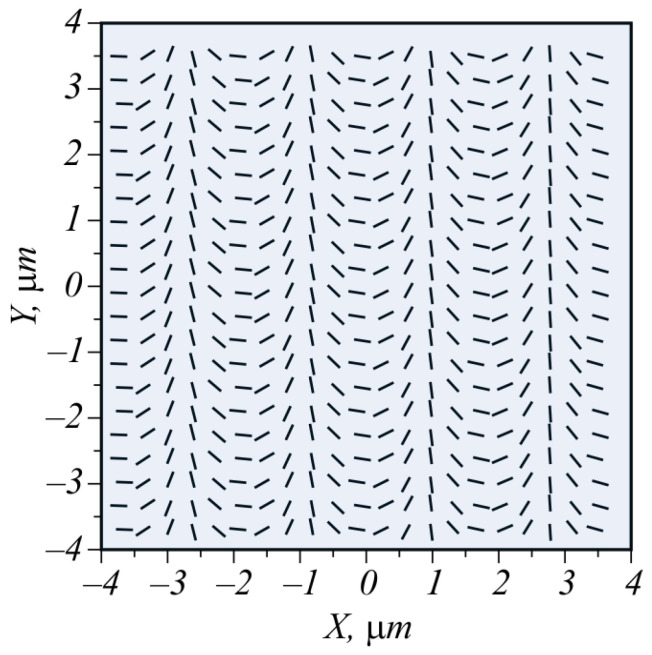
Direction of linear polarization in the light field of (19).

Shown in Figure 4 is the polarization of the beam (19) at α = π/2 (in inverse microns, since *x* is measured in μm).

**Figure 5:**
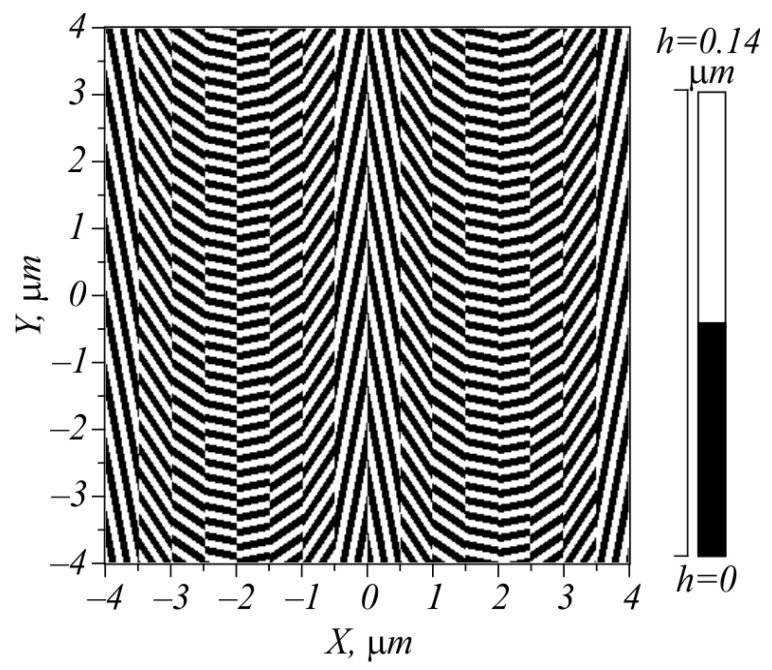
Binary metasurface relief.

Figure 5 depicts a metasurface relief for implementing the transformation matrix $\hat{R}(x)$ of (26) at α = π/2 (in inverse microns).

The metasurface in Figure 5 has a period of 4 μm. The size of the whole metasurface chosen was 8 × 8 μm. It was computed for the wavelength of the incident light, λ = 633 nm. The metasurface is composed of stripes with diffraction gratings with a subwavelength period of 220 nm (the groove width of 110 nm and the step width of 110 nm), rotated by the angle *x*α/2 + π/2 with respect to the axis *x*. Each period is split into 8 stripes, where the angle of the diffraction gratings is constant. The polarization vector of the light field rotates by an angle equal to the doubled angle of rotation of grating lines, which is seen from the comparison of Figure 4 and Figure 5. The relief height is 140 nm, and the refractive index of the gratings is *n* = 4.352 + 0.486*i* (amorphous silicon).

**Figure 6:**
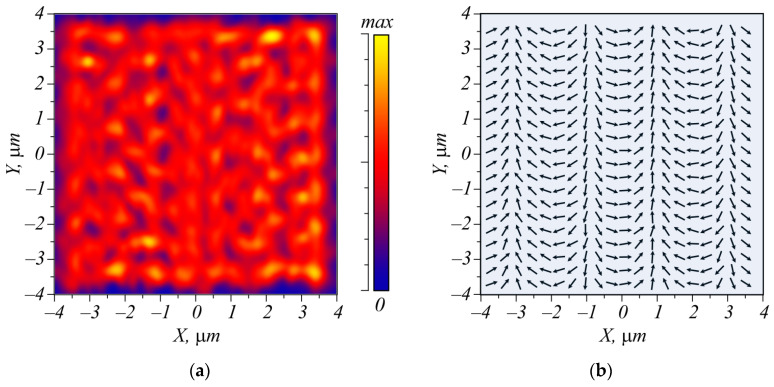
Intensity (**a**) and polarization distribution (**b**) of the electric field at the distance λ from the metasurface.

For the simulation, we used the FDTD method and the Rayleigh-Somerfield transform. At first, we computed by the FDTD method how the light field propagates through the metasurface and obtained the fields *E_x_* and *E_y_* at a distance λ from the metasurface. This field was then the input field of the Rayleigh-Somerfield transform. Using this transform, the resulting light field at a distance of 50 μm was computed. Simulation by only the FDTD method at such a distance (nearly 50 μm) is impossible in 3D due to excessive computational complexity. Shown in Figure 6 is the intensity of the field, obtained at the distance λ beyond the metasurface.

**Figure 7:**
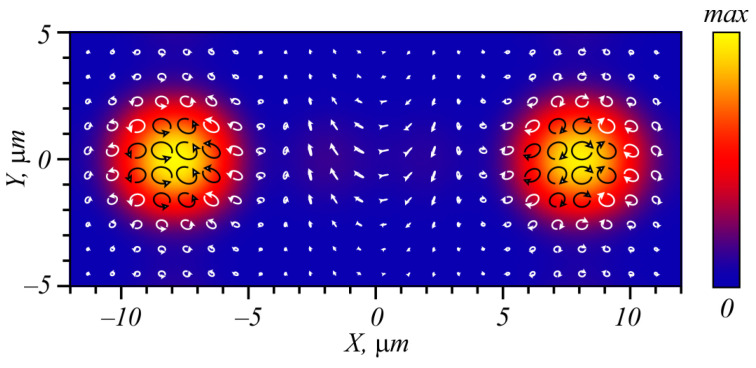
Intensity of light at a distance of 50.633 μm from the metasurface as well as the polarization distribution. Arrows with circles indicate polarization direction in the center of each circle, and the arrow shows the rotation direction of the vector electric field with time.

It is seen that after passing through the diffraction gratings, the field has some inhomogeneity, but nevertheless, the polarization direction is consistent with the distribution obtained by Equation (19) (Figure 4). Figure 7 illustrates the beam intensity at a distance of 50.633 μm from the metasurface, computed by the Rayleigh-Sommerfeld transform.

As seen in Figure 7, at a distance of 50.633 μm from the metasurface, two intensity maxima are generated on the axis *x*, with the distance between their centers being equal to 15.86 μm. In the left intensity maximum, dominating polarization is right-hand circular, while in the right maximum, it is left-hand circular. The distance between the maxima is defined by the period of the metasurface structure (Figure 5. When the metasurface period (Figure 5) doubles, up to 8 μm, the distance between the maxima decreases nearly two times, up to 7.64 μm. From the total energy of the beam coming out of the metasurface, nearly 78.5% goes to both intensity maxima.

**Figure 8:**
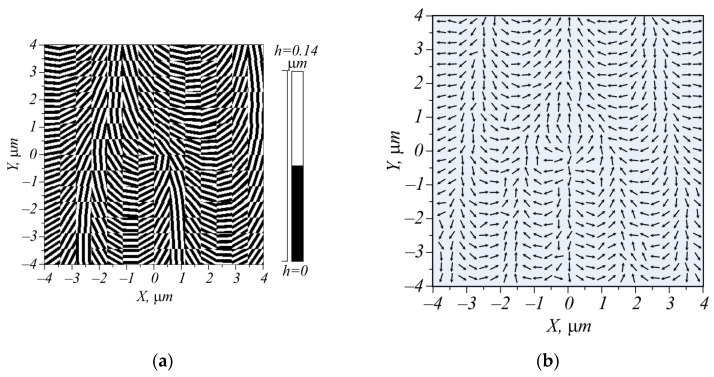
Metasurface, generating the cylindrical vector beam (27) with spatial carrier frequency (**a**), and polarization of a plane linearly polarized wave passed through this metasurface at a distance λ from it (**b**).

Shown in Figure 8a is the metalens that generates the light field (27) with parameters α = π/2 μm^−1^ and *n* = 1. In addition to the spatial frequency, which is present in the metasurface from Figure 5, the topological charge is added here. Therefore, instead of the grating (Figure 5), fork grating was obtained, although only 2 periods were fitted in Figure 8a of such a fork grating (with an edge dislocation). In total, the metasurface with a size of 8 × 8 μm was split into 14 × 14 blocks, each 26 × 26 pixels (0.571 μm).

Shown in Figure 8b is the distribution of linear polarization immediately beyond the metasurface. It is seen that, due to the edge dislocation in the center of the pattern shown in Figure 8b, the polarization singularity, or V-point, appears where linear polarization is indefinite.

**Figure 9:**
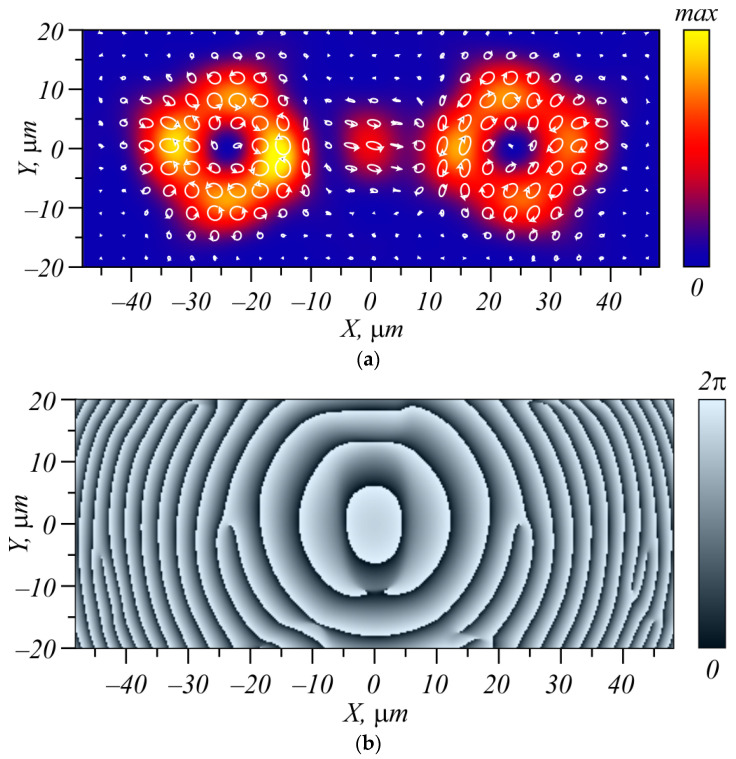
Intensity of the cylindrical vector beam with the carrier frequency, generated by the metalens, and polarization of this beam, depicted as ellipses with arrows (**a**), as well as the phase of the *E_y_* field component (**b**). Each ellipse (**a**) describes rotation of the electric field vector with time.

Figure 9 illustrates the simulation results of light propagation through the metasurface from Figure 8 at a distance of 150 μm from it. In this case, we also first computed the field at a distance of one wavelength from the metasurface by the FDTD method for an exact evaluation of the metasurface contribution. Then, using the Rayleigh-Sommerfeld integral, the field in the far diffraction zone was computed. Since in this case light rings are generated, the propagation distance, where both rings are generated and separated, is larger than in the case shown in Figure 7.

Shown in Figure 9a is an intensity distribution at a 150-μm distance behind the metalens of Figure 8a. It is seen that three light beams are generated: a central one and two light rings to the left and right of the central beam. The intensity of the ring beams is uneven since the metasurface is designed for only two diffraction orders of the grating. Figure 9a also depicts the distribution of the polarization ellipses at a 150-μm distance from the metasurface of Figure 8a. Both rings generated in the far field (Figure 9a) have elliptic and circular polarization—light with right-hand circular polarization generates a ring in the negative part of the *x*-axis (i.e., to the left), whereas light with left-hand circular polarization generates the right ring. In the phase distribution in Figure 9b, forks can be seen near the intensity minima of the two rings. These are optical vortices (screw dislocations) with the topological charges −1 (to the left) and +1 (to the right).

## 8. Conclusions

We have considered two perturbations of a cylindrical vector beam that generate a spin Hall effect, i.e., areas with a nonzero spin angular momentum density occur on propagation, despite a zero spin angular momentum in the initial plane.

The first perturbation was introduced by adding a linearly polarized beam. Thus, we have analyzed the superposition of two rotationally symmetric paraxial vectorial light fields without a spin angular momentum, with one field being cylindrically polarized and the other linearly polarized. The radial distribution of these fields can be arbitrary. Upon separate propagation in free space, these fields do not acquire spin angular momentum and conserve their polarization. The superposition of these fields has inhomogeneous linear polarization in the initial plane, but on propagation in space, polarization becomes elliptic, and thus a nonzero spin angular momentum is generated. The distribution pattern of the spin angular momentum density consists of alternating spots with left- and right-handed elliptic polarization. Such a separation of the light field into areas with opposite-sign spin angular momentum is a manifestation of the optical spin Hall effect. In this work, we discovered and proved two properties of this phenomenon.

The first property is that the transverse shape of the spin angular momentum density distribution does not depend on the weight coefficients of the superposition. The weights define only the magnitude of the spin angular momentum but not the distribution shape.

The second property is that if each such superposition is normalized by its energy, then the greatest values of the spin angular momentum are achieved when both constituent beams have the same energy, i.e., one half of the whole beam energy is the energy of the cylindrically polarized field and the other half is the energy of the linearly polarized field.

The second perturbation of a cylindrical vector beam was introduced by adding a spatial carrier frequency. Upon free-space propagation, such a beam has been shown to split into two shifted off-axis vortex beams, one with right-hand and the other with left-hand circular polarization. This is also a manifestation of the spin Hall effect.

Such a field has been generated by using a metasurface implemented in a thin amorphous-silicon film designed for a wavelength of 633 nm. The metasurface is composed of 14 × 14 blocks of binary subwavelength gratings with a period of 220 nm, which periodically change their direction. A linearly polarized light field incident onto the metasurface was converted into two vortex beams diverging at a certain angle, with one beam being left-handed elliptically polarized and the other right-handed elliptically polarized. The diffractive efficiency of such a grating was found to be almost 80%. These are the simplest metasurfaces for efficiently generating the spin Hall effect in paraxial laser beams. A similar splitting of light beams with opposite-sign spins was implemented by a polarization converter [36].

The discovered properties can be used to enhance the spin angular momentum of light fields. In optical trapping, their use should increase the efficiency of making trapped particles rotate around their centers of mass [37,38]. In optical data transmission, the results obtained can increase the signal power when the data are encoded in light beams by their polarization, and then the incoming beams are identified by polarizers [39,40]. In addition, the obtained results can be used for processing polarization-sensitive materials [41].

## Data Availability

Data are contained within the article.
